# Clinical inter‐rater reliability of postural control techniques

**DOI:** 10.1002/cre2.38

**Published:** 2016-08-11

**Authors:** Ken Yuasa, Yoshiaki Ihara, Yoshiko Takei, Michael E. Groher, Koji Takahashi

**Affiliations:** ^1^ Department of Special Needs Dentistry, Division of Oral Rehabilitation Medicine Showa University School of Dentistry Tokyo Japan; ^2^ Truesdail Center for Communicative Disorders University of Redlands California United States

**Keywords:** dysphagia, postural control techniques, range of motion measurement, Youden plot

## Abstract

Effectiveness of postural control techniques to compensate for oropharyngeal dysphagia have been recommended and used by several clinicians. However, the inter‐rater reliability of these techniques is not well understood. The purpose of this study was to clarify the ambiguity of postural control techniques using statistical analyses. A total of 50 clinicians involved in dysphagia treatment participated in this study, where a healthy male served as the simulated patient. The following clinically used postures were measured by two investigators on two separate days: chin down, right/left incline, and right/left rotation. Postural angles were measured twice by two investigators on each day. Data obtained for the angle of each posture were visually displayed. Data from both investigators were assessed for each posture using the Youden plot, which analyzes data variability for systematic errors and accidental errors separately. The correlation coefficient for examining the measurement error between investigators was calculated. The results showed considerable variation between clinicians regarding the postures used, and significant differences were noted each day. The correlation coefficient for a total of four measurements was more often lower on Day 2 than that on Day 1. The details of the instructions provided by clinicians were not fixed, and the same specified posture was not reproduced even when instructions were provided to the same subject. These findings suggest poor inter‐rater reliability because of the variability of selected postures when using statistical analyses. Therefore, standardized postures need to be developed that can be easily measured and reproduced.

1

In the treatment of patients with dysphagia, compensatory techniques and rehabilitation strategies are used. Compensatory techniques are designed to make swallowing safer and more efficient (Ohmae et al., 1997; Solazzo et al., 2012). These include swallowing maneuvers, multiple swallows, dietary modifications, and postural control techniques.

Postural control techniques are designed to reduce aspiration and penetration by changing the angle and position of the head and body (Ertekin et al., 2001; Logemann, Kahrilas, Kobara, & Vakil et al., 1989; Shanahan et al., 1993). Depending on the specific swallowing deficits found in a patient with dysphagia, a single posture or combination of postures is chosen to facilitate efficient and safe swallowing (Ota, Saitoh, & Matsuo et al., 2002). Effectiveness of postural control techniques has been studied by many clinicians and investigators (Logemann, Rademaker, & Pauloski et al., 1994) since Larsen (Larsen, 1973) recommended the flexed neck posture in 1973. The clinical benefit of applying a single postural control technique or a combination of several techniques has been reported to be effective in 80% to 90% of patients (Logemann et al., 1994; Fujishima et al., 2010). Logemann described these techniques based on videofluorographic data in a significant number of patients with dysphagia (Logemann, 1983). Postural control techniques improve dysphagia by altering the configuration of the oral cavity or pharynx, in an attempt to redirect the bolus or to change the speed of bolus flow (Logemann et al., 1989). Clinicians involved in dysphagia treatment should thoroughly understand how each posture impacts swallowing physiology before selecting a specific posture.

The use of certain postural control techniques is a challenge, as these are not clearly defined. Therefore, postural control techniques currently in use may not be standardized across clinicians. Additionally, there are no studies with statistical data reporting the reproducibility of these techniques. The criteria used to assume a correct posture (such as the angle of inclination of the body) remain ambiguous. The purpose of this study was to provide objective data about inter‐rater reliability of postural control techniques practiced by clinicians familiar with postural controls using statistical analyses.

## 2 PARTICIPANTS AND METHODS

### 2.1 Participants

This study was approved by the Ethics Committee of Showa University School of Dentistry (Approval No. 2013‐004).

Before the initiation of the study, written informed consent was obtained from all participants. A total of 50 clinicians (48 dentists and two speech‐language therapists) involved in dysphagia rehabilitation participated in this study. The years of the experiences were 1 through 17. It was verified that each clinician used postural control techniques in their practice.

A healthy male volunteer (26 years of age; BMI 19.0) served as the simulated patient, and it was confirmed that he had no history of orthopedic disease, abnormalities of cervical alignment, or abnormal findings such as muscular spasms, which may affect adjustment of the instructed posture. The simulated patient wore the same clothes throughout each measurement. Additionally, he wore a swimming cap to reduce systematic error due to hair and a singlet to allow easy assessment to the upper body and cervical region.

After the simulated patient was placed in the targeted posture, the range of motion angles was independently measured by two investigators (one male and one female dentist).

### 2.2 Measurements

**Figure 1:**
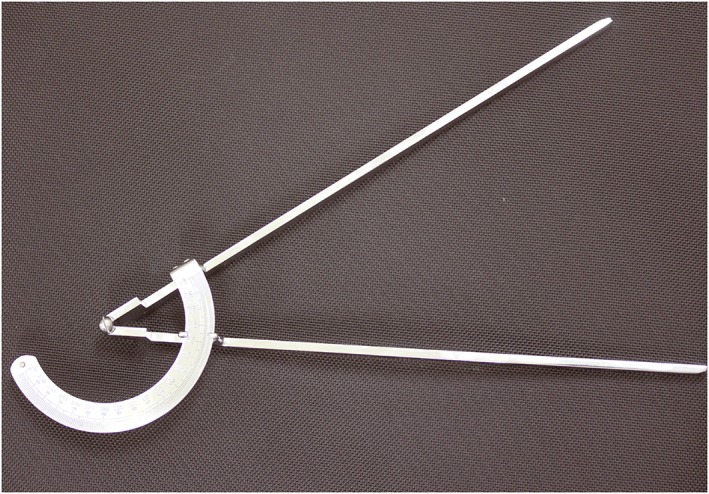
Instrument used for measurement. A University of Tokyo‐style angle gauge (1° increments, Yasuda Ltd. Tokyo, Japan).

**Figure 2:**
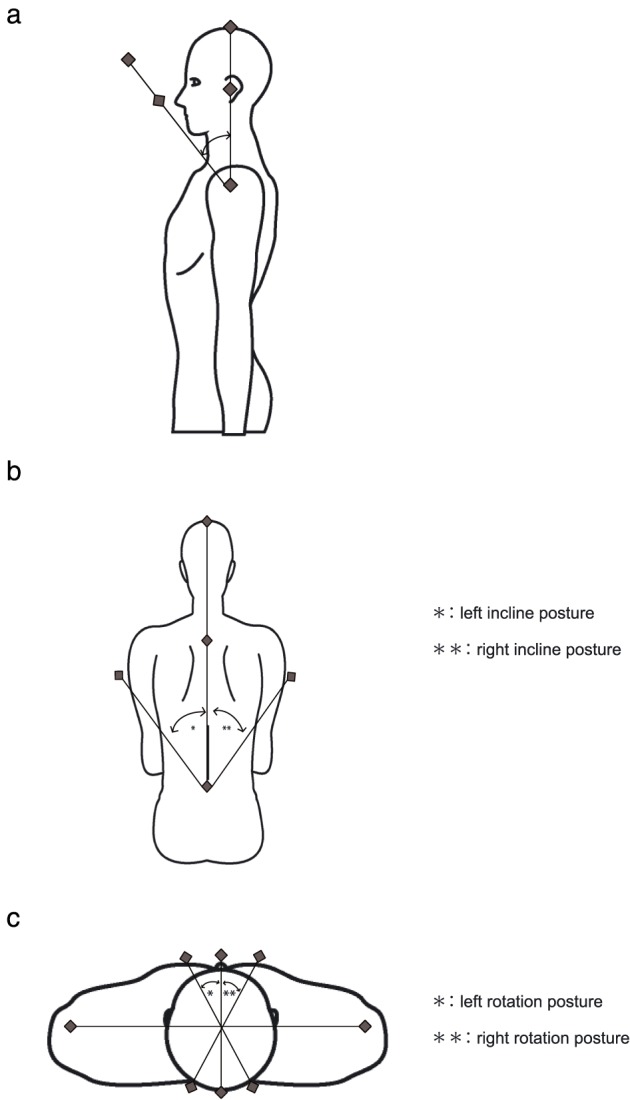
Measurement angles. (a) The angle of chin‐down posture: Angle between the targeted line in the upright sitting position and that in the adjusted posture was measured; targeted line is the line connecting the top of head, ear opening, and the acromion; and rotation axis is the line connecting the acromion of both sides, which is the vertical to the targeted line. (b) The angle of right/left incline posture: Angle between the targeted line in the upright sitting position and that in the adjusted posture was measured. *, left incline posture; **, right incline posture. Targeted line is the vertical line stood at the midpoint of the Jacoby line and rotation axis is the sagittal line passing through the center of the fifth lumbar spinous. (c) The angle of right/left rotation posture: Angle between the targeted line in the upright sitting position and that in the adjusted posture was measured. *, left rotation posture; **, right rotation posture.Targeted line is the vertical line passing through the midpoint of a line connecting the acromion on both sides. This line is equal to that passing though the occipital apex and nasal apex. Rotation axis is the coronal line passing though the midpoint of the line connecting the acromion on both sides, which is equal to the vertical line from the floor passing though the line connecting the occipital apex and nasal apex.

The University of Tokyo‐style angle gauge (1° increments, Yasuda Ltd.) was used for measurement (Figure 1; Imai & Maruyama, 2011) Each investigator read the gauge and recorded the reading after the simulated patient was placed in position. Measurements were performed according to the postures defined in “Measurement Angle of the Range of Motion Joints” (Jpn. J. Rehabil. Med., [Link]). Chin down or cervical flexion was measured by drawing a line from the head to the auditory meatus. The rotation axis of the chin‐down posture was the line connecting the acromion on both sides. The angle between the targeted line in the upright position and that in the adjusted posture was measured and evaluated as the angle of chin‐down posture (Figure 2a).

Lateral incline (right/left bending) of the upper body was measured in terms of the targeted vertical line at the midpoint of the Jacoby line (the line connecting the highest point of the iliac crest on both sides). The rotation axis of lateral incline of the upper body was a sagittal line passing through the center of the fifth lumbar spinous process. The angle between the target line at upright sitting position and target line at the adjusted posture was measured and evaluated as the angle of lateral incline posture (Figure 2b).

Cervical rotation (right/left bending) was measured by drawing a vertical line passing through the midpoint of the line connecting the acromion on both sides. This line is equal to a line passing through the occipital apex and nasal apex. Rotation axis of the cervical rotation was considered as a vertical line from the floor passing through the midpoint of a line connecting the acromion on both sides. The angle between the targeted line at the start of the upright sitting position and that at the adjusted posture was measured and evaluated as the angle of cervical rotation posture (Figure 2c).

### 2.3 Procedure

Each clinician applied the postural control techniques displayed on the monitor to the simulated patient. Only the name of each posture was displayed on the monitor: chin down, right and left incline of the upper body, and right and left cervical rotation. Each clinician placed the simulated patient in the position they saw on the monitor. Once they were satisfied that the posture was correct, they raised their hand, and the investigators came into the room to take measurements of the posture (See sections 1.2 Measurements). The measurements were performed twice by each investigator and were not shared. The identical experiment was conducted the following day. Therefore, each investigator performed two sets of measurements each day, using five different postures.

### 2.4 Data analysis

We obtained the correlation coefficient to examine measurement error between the two investigators. Data on the angle obtained for each posture were visually displayed, and data from both investigators were assessed for each posture, using the Youden plot, which separately displays data variability for systematic errors and accidental errors (Skendzel & Youden, 1970; Skendzel & Youden, 1969; Youden, 1977). Data from the investigators were assessed for the first and second measurements for each day of the study.

## 3 STATISTICAL ANALYSES

**Figure 3:**
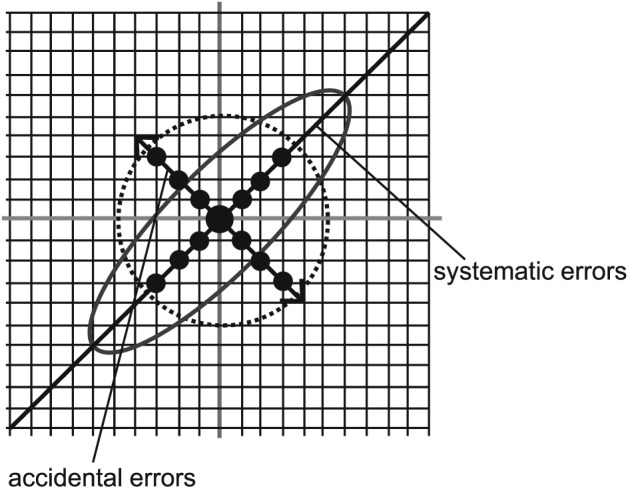
Components of Youden graph. Youden plots were obtained for each adjusted posture. The angle obtained for each posture was visually displayed in the Youden plot, which can visually indicate systematic errors and accidental errors, separately. The Youden plot shows bias in terms of systematic errors (presented as an ellipse in the direction from the bottom left to the top right) and accidental errors (presented orthogonally to systematic errors from the top left to the bottom right). In the Youden plot, the 95% confidence interval was elliptical, and systematic errors were noted. ●, plot.

The Youden plot analysis is a scatter diagram used to determine the precision of laboratory data collection. It is prepared by plotting one variable on the abscissa and the other on the ordinate to visually display each data point. Youden plots can show bias in terms of systematic error (presented as an ellipse in the direction from the bottom left to the top right) and accidental errors (presented orthogonally to systematic errors from the top left to the bottom right). The size of the ellipse indicates magnitude of bias. If there is no correlation between the two sets of data, bias is represented by a circular distribution expressed with broken lines. Using this model, a large amount of bias is consistent with poor reproducibility of the instructions provided by the clinicians, while limited bias shows the converse. In this manner, the effectiveness of the required instructions provided by an instructor can be assessed based on their reproducibility (Figure 3). Additionally, we analyzed the angle of each posture between Day 1 and Day 2 using Spearman's rank correlation coefficient. Spearman's rank correlation coefficient, nothing without assumptions about the distribution of the two variables, one in which the relationship between the variables to assess how well faithfully represented by any monotonic function. This statistic analyses can determine measurements of the angle of each posture of Day 1 and Day 2 reproducibility how match. The level of significance was defined as *p* < .01. Statistical analysis was performed using JMP Pro version 12 software.

## 4 RESULTS

**Figure 4:**
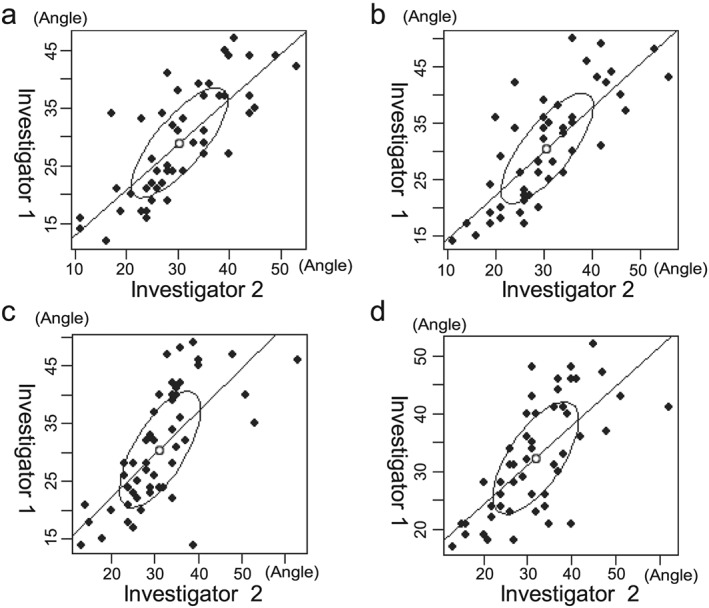
Result measuring angle of the chin‐down posture. Angles measured by Investigator 2 were corresponding to the X value. Data of angles measured by Investigator 1 corresponded to the Y value. A point that represented the intersection of the results of two investigators was plotted. The elliptical shape indicates the 95% confidence interval, while the range of the elliptical shape in the direction from the bottom left to the top right indicates the degree of systematic error. ●, plot; ○, median; /, correlation coefficient. Day 1: (a) the correlation coefficient for the first measurement was 0.78 and (b) the correlation coefficient for the second measurement was 0.76. Day 2: (c) the correlation coefficient for the first measurement was 0.69 and (d) the correlation coefficient for the second measurement was 0.68. This result represents poor reproducible of chin‐down posture caused by bias from subjective evaluations by the clinicians.

The results for the angle of the chin‐down posture are shown in Figure 4. The correlation coefficients between the two investigators for the first and second measurement on Day 1 were moderately high (0.78 and 0.76, respectively) while those on Day 2 was 0.69 and 0.68, respectively. The average angle on Day 1 was 28.98° and that on Day 2 was 30.68° (*p* = .69, Table 1).

**Table 1:** The results for each posture two investigators for the first and second measurement on Day 1 and Day 2

Postures (°)	Day 1 (*n* = 50)	Day 2 (*n* = 50)	*p*
Chin down	28.98 ± 10.60	30.68 ± 9.59	.69
Right incline	12.54 ± 8.03	13.52 ± 6.40	.57
Left incline	10.86 ± 6.30	11.78 ± 6.16	.78
Right cervical rotation	46.12 ± 27.79	45.33 ± 24.32	.58
Left cervical rotation	56.67 ± 28.76	54.63 ± 25.29	.71

*Note*. Spearman's rank correlation coefficient.

**Figure 5:**
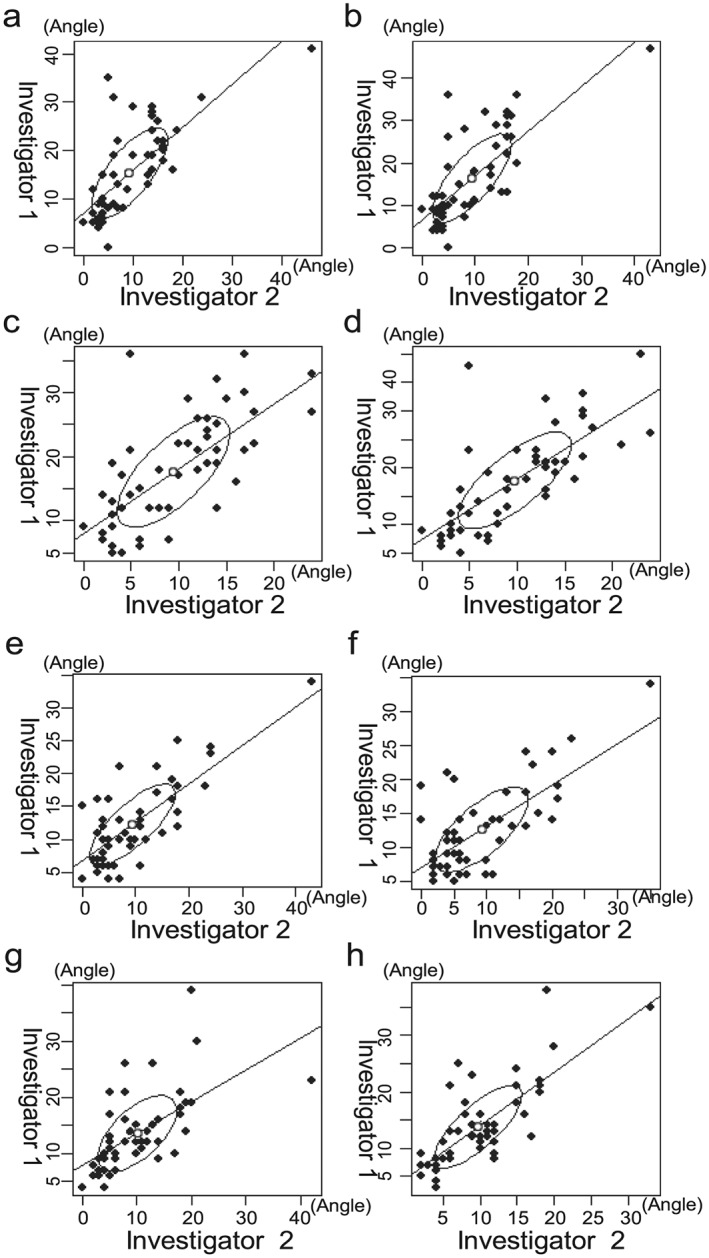
Result measuring angle of the right/left incline of upper body posture. Youden plots were obtained for the right/left incline of upper body postures. ●, plot; ○, median; /, correlation coefficient. Right incline posture: Day 1: (a) the correlation coefficient for the first measurement was 0.72 and (b) the correlation coefficient for the second measurement was 0.75. Day 2: (c) the correlation coefficient for the first measurement was 0.70 and (d) the correlation coefficient for the second measurement was 0.74. Systematic errors were indicated in the Youden plot for the right incline posture. Left incline posture: Day 1: (e) the correlation coefficient for the first measurement was 0.76 and (f) the correlation coefficient for the second measurement was 0.70. Day 2: (g) the correlation coefficient for the first measurement was 0.61 and (h) the correlation coefficient for the second measurement was 0.77. Systematic errors were indicated in the Youden plot for the left incline posture.

The results for the angle of the right incline of the upper body posture are shown in Figure 5a–d. The correlation coefficients between the two investigators for the first and second measurement on Day 1 were 0.72 and 0.75 while those on Day 2 were 0.70 and 0.74, respectively. The average angle on Day 1 was 12.54° and that on Day 2 was 13.52° (*p* = .57, Table 1 ).

The results for the angle of the left incline of upper body posture are shown in Figure 5e–h. The correlation coefficients between the two investigators for the first and second measurement on Day 1 were 0.76 and 0.70 while those on Day 2 were 0.61 and 0.77, respectively. The average angle on Day 1 was 10.86° and that on Day 2 was 11.78° (*p* = .78, Table 1).

**Figure 6:**
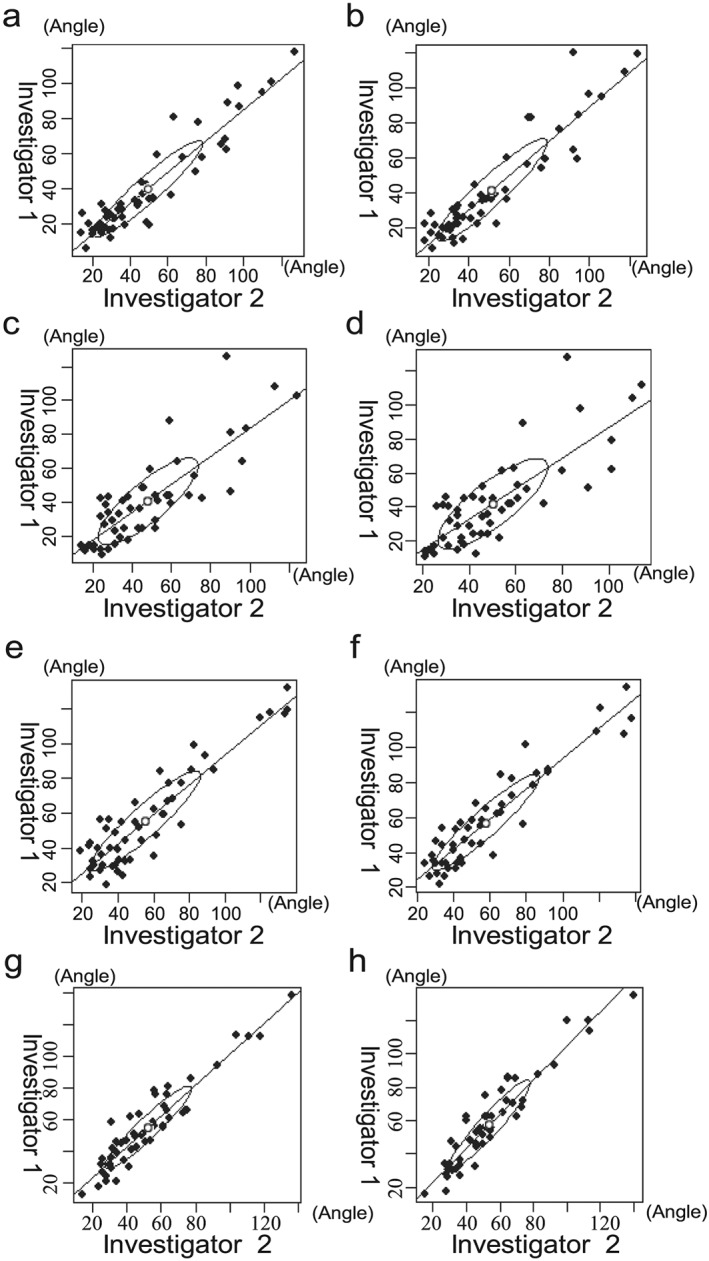
Result measuring angle of the right/left cervical rotation posture. Youden plots were obtained for the right/left cervical rotation postures. ●, plot; ○, median; /, correlation coefficient. Right rotation: Day 1: (a) the correlation coefficient for the first measurement was 0.94 and (b) the correlation coefficient for the second measurement was 0.93. Day 2: (c) the correlation coefficient for the first measurement was 0.83 and (d) the correlation coefficient for the second measurement was 0.81. Systematic errors were indicated in the Youden plot for the right rotation posture. Left rotation: Day 1: (e) the correlation coefficient for the first measurement was 0.92 and (f) the correlation coefficient for the second measurement was 0.93. Day 2: (g) the correlation coefficient for the first measurement was 0.94 and (h) the correlation coefficient for the second measurement was 0.94. Systematic errors were indicated in the Youden plot for the left rotation posture.

The results for the right cervical rotation posture are shown in Figure 6a–d. The correlation coefficients between the two investigators for the first and second measurement on Day 1 were 0.94 and 0.93 while those on Day 2 were 0.83 and 0.81, respectively. The average angle on Day 1 was 46.12° and that on Day 2 was 45.33° (*p* = .58, Table 1).

The results for the left cervical rotation posture are shown in Figure 6e–h. The correlation coefficients between the two investigators for the first and second measurement on Day 1 were 0.92 and 0.93 while those on Day 2 were 0.94 and 0.94, respectively. The average angle on Day 1 was 56.67° and that on Day 2 was 54.63° (*p* = .71, Table 1).

On the Youden plot, the 95% confidence interval was elliptical, and systematic error was noted for all measurements plotted. This result represents poor reproducibility because of the variability of selected postures. Nevertheless, no significant difference was noted between the angle of each posture on Days 1 and 2.

## 5 DISCUSSION

The settings for patients with dysphagia include hospitals (Cherney, 1994), nursing homes (Siebens et al., 1986), or their own homes (Lindgren & Janzon, 1991), and theoretically, the precise compensation used for swallow safety must be determined for each patient. To accurately reproduce the specified posture, various media, such as verbal communication, written documents, illustrations, photos (still images), and videos (moving images), may be used in clinical practice. In such settings, reproducibility of postural control techniques might be lower than that in a physician's office. In this study, the Youden plot showed considerable variation in the measurement data. It is assumed that considerable variation due to bias would occur when an angle gauge is used to measure the range of motion in the clinical setting. However, there are no studies using statistical analysis that report the potential variance in assuming the correct posture. In contrast, in this study, findings are shown using Youden plots and correlation coefficients. In addition, we used Spearman's rank correlation coefficient. The presence of bias (postural variation) could be related to the investigator's expertise in postural control, the vagueness of evaluation terminology, and/or variation in the comprehension of instructions provided by the clinicians. It was expected that the correlation coefficient on Day 2 would be higher than that of Day 1 because the experience was replicated. However, the correlation coefficient for a total of four measurements was more often lower on Day 2 than that on Day 1. Additionally, a large value of standard error was observed for the same posture. This finding indicated that details of the instructions provided by clinicians were not standardized, allowing for variations in measured postural angles on different days, even by the same clinician. Thus, the same specified posture was not reproduced even when instructions were identical. This finding suggests that the methods of posture assessment (the definition of posture measurement) and measurement (with an angle gauge), which are presently employed in the clinical setting, are inadequate to achieve high reproducibility. Additionally, it is suggested that repetitive experience does not improve reproducibility of postural positions. Owing to such factors, the postural control techniques may be difficult to accurately replicate over repetitive sessions during dysphagia treatment. Therefore, taking the definition and assessment of postural control techniques as it is currently performed clinically into account, reproducibility is considered to be inadequate for clinical application to dysphagia rehabilitation. The angle of posture for the swallowing function has a significant impact (Ota, Saitoh, Kagaya, Sonoda, & Shibata et al., 2011; The Japanese Society of Dysphagia Rehabilitation, 2014). Therefore, a highly reproducible method of postural control that is independent of experience is required.

The effectiveness of postural control techniques is stated in the “Summary of Training Methods” (Fujishima et al., 2010). However, there are no systems available to measure and confirm whether the specified posture adjustment is actually reproducible. Furthermore, craniocervical position is assessed while the trunk is in a fixed posture during dysphagia rehabilitation. As a result, only the distal parts of the body are assessed without considering the position of the trunk, which is essential for postural control techniques. Whether the posture achieved by postural control techniques is reproduced correctly or not needs further investigation as it can easily be influenced by the clinician's bias due to variation in the precision of measurement.

To assess the reproducibility of posture, several devices such as specialized training chairs and head/neck fixing apparatus have been developed (Logemann, 2008). However, no method is available to evaluate the patients' postures while using these devices. It is also important to evaluate postural controls during actual swallowing as the conditions may change during the patient's attempt to swallow. In this study, we measured the reproducibility of postural control techniques conducted by clinicians. In the future, we need to study the potential differences between a posture set by the clinician and the actual posture the patient is in at the moment of swallowing. Establishing the most reliable measuring tool is also of interest for future study.

A limitation of this study was that only two‐dimensional (*x* and *y*) measurements were taken, where there is a need to examine three‐dimensional changes including the trunk of the body (*x*, *y*, and *z*) as a large number of patients with dysphagia consume their meals while being seated on a chair. In this case, the posture is affected in three dimensions. This was a pilot study to determine the inter‐rater reliability of postural control techniques using statistical analyses, and we used only one simulated patient. Further study involving patients with different physiques, physical characteristics, and age is required.

## CONFLICT OF INTEREST

None of the authors has any conflicts of interest to declare with respect to this study.
